# Idiopathic hypereosinophilic syndrome presenting with liver mass: Report of two cases: Idiopathic hypereosinophilic syndrome and liver mass

**Published:** 2011-02-01

**Authors:** Kamran shatery, Alireza Sayyah

**Affiliations:** 1 Department of Internal Medicine, Urmia University of Medical Sciences, Urmia, IR Iran

**Keywords:** Hypereosinophilic syndrome, Liver

## Abstract

Herein, we report on two cases of hypereosinophilic syndrome presenting as liver mass. One patient was a 22-year-old woman presented with fever, upper abdominal pain, nausea/vomiting and a mass in the right liver lobe. The second patient was a 54-year-old man who presented with nausea and abdominal pain with significant weight loss. He had multiple lesions in both liver lobes. Both patients had eosinophilia that was not attributed to other causes such as allergy or parasites. The patients were treated with glucocorticosteroid and improved clinically. Imaging and laboratory abnormalities resolved.

## Background

The hypereosinophilic syndromes (HES) are disorders marked by the sustained overproduction of eosinophils, often but not invariably, associated with damage to multiple organs due to eosinophilic infiltration and mediator release [1][2][3][4]. The diagnosis of HES should be suspected in patients with sustained blood eosinophilia of ≥1500/µL, documented on at least two occasions, no other apparent etiologies for eosinophilia, and often signs and/or symptoms of end-organ dysfunction. Hepatic involvement may take the form of chronic active hepatitis, focal hepatic lesions, eosinophilic cholangitis, or the Budd-Chiari syndrome. Manifestation of HES as liver mass is rare. Herein we report on two cases of HES presenting with liver mass.

## Case 1

**Figure 1.**
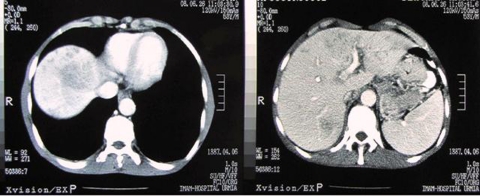
Note the mass lesion in the right liver lobe on abdominal CT performed with intravenous contrast

A 22-year-old Caucasian woman with unremarkable past medical history, presented with fever, followed by loss of appetite, epigastric and right upper quadrant pain. She also complained of nausea and vomiting and 2-kg weight loss over one month period. She reported no change in bowel habit, no pruritus or icter. She lived in West Azarbaijan and denied any history of international travelling. Past history for asthma, allergy, allergic rhinitis was negative. Drug history was negative except for H2-receptor antagonist and metochlopramide which she took during last month for her recent gastrointestinal symptoms. Her vital signs were within normal range. The only physical finding was epigastric tenderness. In initial assessment, she had a leukocytosis of 17,870/µL with 19.6% neutrophils, 18.8% lymphocytes, 56.1% eosinophils, and 1.2% basophils; she had a hemoglobin of 12.4 mg/dL and a platelet count of 433,000/µL (Table 1). Liver function tests and electrolytes were normal. Erythrocyte sedimentation rate (ESR) was elevated up to 65 mm/h. C-reactive protein (CRP) was 3+ positive. Urinalysis was normal. Stool examination for ova/parasite was negative in three consecutive times. Serology tests for Fasciola, visceral larva migrans (Toxocara canis), and hydatid disease (Ecchinococcus granolosus) were negative. An abdominal ultrasound revealed a hypoechoic liver mass with irregular borders and internal amorphous echogenic areas in addition to multiple ill-defined hypoechoic lesions in right liver lobe. Computed tomography (CT) confirmed a hyperdense mass (Figure 1). Esophagogastroduodenoscopy was normal. Chest radiography was normal. Serologic markers for chronic viral hepatitis were negative and α-fetoprotein was normal. Ultrasound-guided liver biopsy was done which revealed portal mixed neutrophilic and lymphocytic infiltrates, stellate (interface) hepatitis, and vague nodule formation but no eosinophilic infiltration. Pathology was graded as "mild to moderate chronic hepatitis". Bone marrow biopsy showed normocellular polymorphic marrow with increased eosinophilic series. She was treated with oral prednisolone, 50 mg daily that was tapered over three months and remained asymptomatic after six months; liver ultrasound and CT became normal and there was no more eosinophilia on peripheral blood film.

**Table 1:** Laboratory findings at presentation

**Test results**	**Patient 1**	**Patient 2**
**WBC **(No/µL)	17870	16000
**Polymorphonuclear **(%)	19.6	21.4
**Lymphocyte** (%)	18.8	4.5
**Eosinophil** (%)	56.1	69
**Basophil **(%)	1.2	1
**Monocyte** (%)	2.6	3.3
**Hemoglobin** (mg/dL)	12.4	10.4
**Platelet **(No/µL)	433,000	273,000
**ESR** (mm/h)	65	40
**CRP**	3+	3+
**PT** (s)	13	14
**PTT** (s)	28	46
**ALT **(U/L)	18	50
**AST** (U/L)	13	61
**ALKP**	163	463
**Bilirubin T–D** (mg/dL)	0.61–0.20	0.58–0.19

## Case 2

The second patient was a 53-year-old Caucasian man with 20 days history of fatigue, weakness, and steady right upper quadrant pain, not related to eating. He had nausea without vomiting. He reported 14-kg weight loss during this short period. He lived in West Azarbaijan and denied history of international travelling. Past history for asthma, allergy and allergic rhinitis was negative. Drug history was also negative. Physical examination was insignificant except for an epigastric tenderness in deep palpation. In initial assessment, he had leukocytosis of 16,000/µL (21.4% neutrophils, 4.5% lymphocytes, 69% eosinophils, 1% basophils, and 3.3% monocytes). He had a hemoglobin concentration of 10.4 mg/dL and a platelet count of 273,000/µL (Table 1). He had elevated aminotransferase levels less than two times upper normal limit. Electrolytes were normal. ESR was elevated up to 40 mm/h. CRP was 3+ positive. Urinalysis was normal. Schistosomiasis was ruled out. Stool examination for ova/parasite for three consecutive times was negative. Serology tests for Fasciola, visceral larva migrans (Toxocara canis), and hydatid disease (Ecchinococcus granolosus) was negative. An abdominal ultrasound was normal. CT of abdomen with intravenous and oral contrast showed multiple diffused 5-10-mm lesions in both liver lobes. Esophagogastroduodenoscopy and colonoscopy were normal. Chest radiography was normal. Bone marrow aspiration and biopsy showed a hypercellular marrow with polymorphic cell population and increased number of eosinophilic series. Liver biopsy was diffusely infiltrated by mixed inflammatory cells, predominantly clusters of eosinophils with liver parenchymal destruction in some areas. He received oral prednisolone 30 mg daily which was tapered over six months and regained his lost weight. He is symptom-free now and eosinophilia has resolved. Also anemia was corrected by the treatment. A control CT of liver 12 months later, showed resolution of liver lesions.

## Discussion

HES is most common in patients 20 to 50 years of age [4]. The FIP1L1-PDGFR alpha-associated variant affects mostly males, whereas other variants appear to affect males and females equally. Patients with HES may present with insidious and incidentally detected eosinophilia, gradually progressive symptoms, or with life-threatening cardiac and neurologic events such as thromoembolic stroke or encephalopathy. The organ systems most commonly affected in HES are the heart, nervous system, skin, lungs, and gastrointestinal tract [5]. Fatigue, cough, breathlessness, muscle pains, angioedema, rash, fever, involvement of the heart, skin, nervous system, lungs, and spleen, each occurs in 40% to 64% of patients; liver, sinus, ocular, and gastrointestinal involvement are less common (14% to 32% each) [4][6]. Gastrointestinal manifestations include eosinophilic gastritis, enteritis, and/or colitis causing weight loss, abdominal pain, vomiting, and/or severe diarrhea [4]. Hepatic involvement may take the form of chronic active hepatitis, focal hepatic lesions, eosinophilic cholangitis, or the Budd-Chiari syndrome. History for allergic disorders, medications, and travelling should be sought and patients should be investigated for helminthic/parasitic infections. Echocardiography and serum troponin level measurement should be done to monitor for cardiac involvement. Liver biopsy should be done in case of hepatic involvement to make a definite diagnosis and a bone marrow biopsy to rule out myeloproliferative disorders, mastocytosis, acute and chronic eosinophilic leukemia, etc. [7][8][9][10][11]. These workups however, did not uncover end-organ involvements in our patients except for liver damage. Both patients responded well to glucocorticosteroid therapy. Both patients are on low-dose steroid and monitored for symptoms and eosinophilia on follow-up visits. Presentation of HES as liver mass is rare; however, we reported two patients without any other end-organ involvement. Our patients differed not only in gender and age but also in the pattern of liver involvement; the first patient had a hypodense mass in the right lobe while the second patient had multiple small diffused lesions. Oh, et al. have previously reported a case with single liver mass [12]. Lai, et al. also reported a 52-year-old woman presenting with recurrent abdominal pain and liver masses [13]. In contrast to our second patient, the first one showed no eosinophilic infiltration in liver pathology. Similar observation has also been reported by Minola, et al. [14]. Minola reported a 28-year-old woman, affected by idiopathic HES with bone marrow and pulmonary eosinophilic infiltrates associated with peripheral eosinophilia and features of chronic hepatitis without a significant eosinophilic component. This finding may support the hypothesis that liver damage in idiopathic HES may be due to circulating substances produced by eosinophils rather than direct infiltration of liver by them. Unfortunately, we did not perform IgE assay for our patients that is usually elevated in such cases.In conclusion, HES is a diagnosis that should be considered in liver masses accompanied by peripheral blood eosinophilia. Liver biopsy, bone marrow aspiration and biopsy, and helmintic and parasitologic laboratory tests are usually needed to establish the diagnosis. These patients usually respond very well to corticosteroid therapy.
